# Double deletion of the active zone proteins CAST/ELKS in the mouse forebrain causes high mortality of newborn pups

**DOI:** 10.1186/s13041-020-0557-x

**Published:** 2020-01-29

**Authors:** Akari Hagiwara, Shun Hamada, Yamato Hida, Toshihisa Ohtsuka

**Affiliations:** Department of Biochemistry, Faculty of Medicine, University of Yamanashi, 1110 Shimokato, Chuo, Yamanashi, 409-3898 Japan

**Keywords:** Presynapse, Active zone, CAST, CaMKII Cre, CAZ proteins, Neurotransmitter release

## Abstract

Presynaptic active zone cytomatrix proteins are essential elements of neurotransmitter release machinery that govern neural transmission. Among active zone proteins, cytomatrix at the active zone-associated structural protein (CAST) is known to regulate active zone size in retinal photoreceptors and neurotransmitter release by recruiting Ca^2+^ channels at various synapses. However, the role of ELKS—a protein from the same family as CAST—and the synergistic roles of CAST/ELKS have not been thoroughly investigated, particularly with regard to mouse behavior. Here, we generated ELKS conditional KO in mouse forebrain synapses by crossing ELKS flox mice with a CaMKII promoter-induced Cre line. Results showed that CAST is dominant at these synapses and that ELKS can support CAST function, but is less effective in the ELKS single KO. Pups of CAST/ELKS double KO in the forebrain were born in Mendelian rations but resulted in eventual death right after the birth. Anatomically, the forebrain neuronal compositions of CAST KO and CAST/ELKS double KO mice were indistinguishable, and the sensory neural network from whiskers on the face was identified as barrelette-like patches in the spinal trigeminal nucleus. Therefore, depletion of CAST and ELKS disrupts neurotransmission from sensory to motor networks, which can lead to deficits in exploration and failure to suckle.

## Maintext

Sensory neural networks in the brain pass on information to other systems where it is processed and can then influence behavior. These networks are sustained by numerous excitatory and inhibitory synapses, each of which has pre- and post-synaptic sites. At the presynaptic active zone (AZ), neurotransmitter-containing synaptic vesicles dock close to voltage-gated Ca^2+^ channels and are made ready to fuse with the plasma membrane in a Ca^2+^-dependent manner. To organize the framework of neurotransmitter release, cytomatrix at the AZ (CAZ) proteins, including Munc13, RIM, Bassoon, CAST (also named ELKS2α or ERC2), and ELKS (ELKS1α, CAST2, ERC1) perform a variety of roles such as formation and maintenance of synapses, tethering and docking synaptic vesicles at AZ-release sites, and recruitment of Ca^2+^ channels to the AZ [1–3]. As well as the deletion mutant of each CAZ protein indicated their roles, organization of release site was complemented by differing CAZ proteins, as revealed by combinatorial deletion of proteins, such as ELKS (CAST/ELKS) and RIM, or RIM and RIM-BP, which resulted in a large reduction of docked vesicles and presynaptic dense projections, respectively [4, 5]. In previous reports of the retinal photoreceptor-ribbon synapse, we have shown that CAST functions in AZ formation and the processing of neurotransmission [6]. Intriguingly, although the CAST-family protein ELKS complemented CAST’s functional role with regard to neurotransmitter release, the size of the AZ in CAST/ELKS double KO (dKO) mice was comparable to that of CAST-only KO [7]. The roles of CAST and ELKS in recruiting Ca^2+^ channels to the release site were confirmed at other synapses, including the calyx of Held [8–10]. As a matter of course, the role of CAST in neurotransmission was investigated at the CAST KO hippocampal synapses [11, 12], in which the presynaptic release was increased at excitatory and inhibitory synapses, while the density of synapse and the AZ size was not altered. Analysis of CAST/ELKS dKO in hippocampal primary culture neurons indicated that unlike the RIM binding C-terminal region, the N-terminal region is essential for regulating the readily releasable pool of neurotransmitters [13].

However, the function of CAST/ELKS-related synaptic modulation with regard to behavior has not been well described. To determine any roles of CAST/ELKS on learning, memory and cognition at the cortex and/or hippocampus, we generated CAST KO and ELKS conditional KO (cKO) in the forebrain by crossing CaMKII-Cre mice [14]. As expected, behavior and synaptic connections in the hippocampus were not affected by the ELKS cKO in the forebrain. However, the CAST/ELKS dKO pups surprisingly died right after the birth. Further examination showed that this was despite an intact sensory network system for facial whiskers. Therefore, CAST/ELKS-responsible neurotransmission in the forebrain neural network is essential for newborn mice to acquire normal exploratory or suckling behavior.

## Results and discussion

### Generation of ELKS cKO at the forebrain

**Fig. 1:**
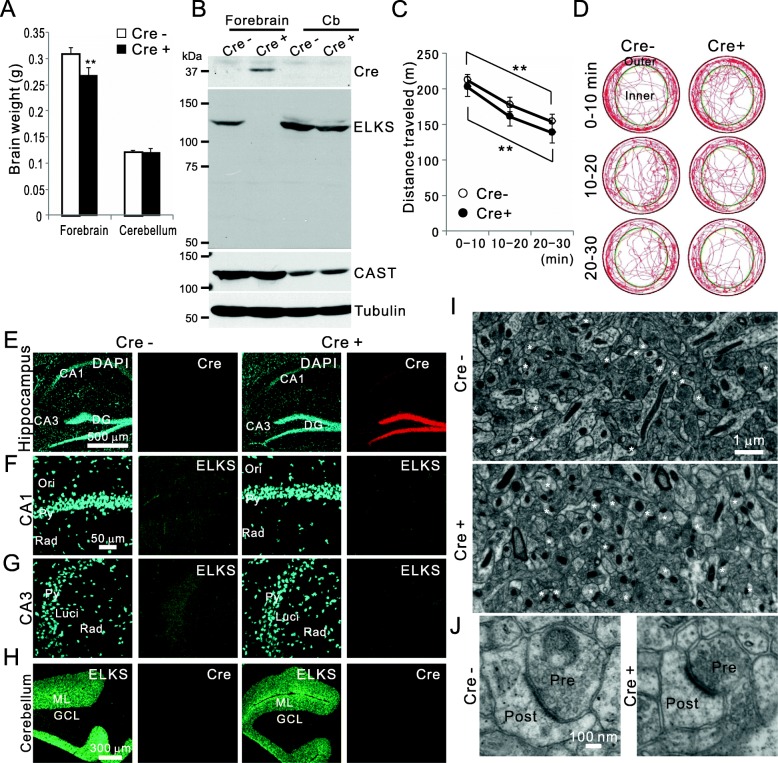
Forebrain specific ELKS cKO by breeding with CaMKII-Cre mouse. **a** Forebrain size in ELKS cKO (ELKS flox; Cre^+^) mice was smaller than in control (ELKS flox; Cre^−^) mice, while the weight of the cerebellum did not differ significantly. Mean ± SD, *n* = 3 for each sample, **P < 0.01 (Student’s t-test). **b** Forebrain and cerebellum (Cb) brain homogenates were analyzed by western blot using the indicated antibodies. Cre recombinase was expressed specifically in the forebrain where ELKS expression was depleted. No truncated ELKS band was detected. **c**-**d** Exploration of novel environment was traced **d** and the distance traveled was measured between 0 and 10, 10–20, and 20–30 min in control (Cre-) and ELKS cKO (Cre+) mice. The distance travelled gradually decreased from 0 to 10 min to 20–30 min, which was not different between control and ELKS cKO mice. **P < 0.01 (one-way ANOVA, post-hoc Tukey’s test). **e**-**g** Immunohistochemistry using the indicated antibodies revealed that Cre expression in the hippocampal dentate gyrus DG granule cells and ELKS expression in the CA1 and CA3 regions was depleted. *Ori*, oriens; *Py*, pyramidale; *Rad*, radiatum; *Luci*, lucidum. **h** ELKS was not depleted in the cerebellum where Cre was not expressed. **i**-**j** Electron microscopy of the hippocampal CA1 stratum radiatum revealed numerous excitatory synapses on the pyramidal cell spines (asterisks). The synapse indicated by the # mark was excluded because mitochondria were present at the postsynaptic site. Presynaptic terminal area and AZ length were measured with high magnification as demonstrated by representative images **j**

To understand the roles of ELKS in forebrain neural networks within the cortex and hippocampus, ELKS flox mice were crossed with Cre knockin mice in which Cre expression was regulated by the CaMKII promoter [7, 9, 14]. Both male and female ELKS cKO mice (ELKS^flox/flox^; CaMKII Cre^+/−^) were viable and fertile, and they exhibited no gross developmental abnormalities, including body weight (Table 1). Weight of the forebrain in these mice was slightly but significantly smaller compared with controls (ELKS^flox/flox^; CaMKII Cre^−/−^), while that of the cerebellum did not differ (Fig. 1a). Cre recombinase targets exon 11 of ELKS and causes a frame shift in exons 12 and 13 [7, 9]. ELKS expression was confirmed by immunoblotting with anti-ELKS antibody raised against amino acid 121–165 located in the first coding exon (exon3), and testing indicated depletion of ELKS in the forebrain of Cre^+^ mice (Fig. 1b). In previous reports, ELKS expression was compensatory enhanced in the forebrain, calyx of Held, and retina of CAST KO mice [9, 12]. Similarly, CAST has been shown to be upregulated in ELKS cKO retinas [7]. In the current study, extensive ELKS depletion in the forebrain did not significantly affect CAST expression, presumably because CAST is dominant at the majority of forebrain synapses—an assumption supported by CAST and ELKS mRNA expression (Allen Brain Atlas). We explored the functional relevance of ELKS in the forebrain by assessing behavior in a novel open field. The distance traveled, and resting time was indistinguishable between control and ELKS cKO mice (Fig. 1c-d, Table 2). Additionally, both control and ELKS cKO mice spent more time traveling along the peripheral zone (Outer) than within the center zone (Inner) (Fig. 1d, Table 2). Immunohistochemistry of the hippocampus confirmed the expression of Cre, especially in the granule cells of the dentate gyrus, following the depletion of ELKS in the CA1 and CA3 regions (Fig. 1e-g). In contrast, ELKS expression was not altered in the cerebellum, where CaMKII promoter-induced Cre was not detected by immunoblotting or immunohistochemistry (Fig. 1b, h). Furthermore, we used electron microscopy to investigate the ultrastructure of synapses in the CA1, which included mostly Schaffer-collateral to pyramidal neuron spines. Neither the density of excitatory synapses nor the size of the preterminal or the AZ significantly differed between ELKS cKO and controls (Fig. 1i-j, Table 3). Similar to a previous report of CAST KO [12], ELKS depletion had little effect on the anatomy of hippocampal synapses.

**Table 1:** Body weight of control and ELKS cKO

	ELKS flox; Cre - (Cont)	ELKS flox; Cre + (ELKS cKO)
Male	24.5 g ± 0.74 (*n* = 4)	22.7 g ± 0.71 (*n* = 7)
Female	20.0 g ± 0.50 (*n* = 5)	20.0 g ± 0.69 (*n* = 5)

Values are means ± SEM. Body weights of mice aged 9–12 weeks were averaged from the sample sizes (n)

**Table 2:** Open field activity in a novel environment (30 min recording) for control and ELKS-cKO mice

Male	n	Distance traveled (m)		Distance (%)	Presence time (min)	Resting time (min)
Cont	6	505.84	Outer area	73.4	23.5	3.23
			Inner area	26.6	6.5	0.57
ELKS cKO	9	458.68	Outer area	72.4	24.4	4.25
			Inner area	27.6	5.6	0.65
Female						
Cont	5	524.62	Outer area	69.8	22.9	3.18
			Inner area	30.2	7.1	0.72
ELKS cKO	5	576.83	Outer area	75.3	25.1	3.51
			Inner area	25.3	5.0	0.44
Total						
Cont	11	514.4	Outer area	71.8	23.2	3.21
			Inner area	28.2	6.8	0.64
ELKS cKO	14	499.6	Outer area	73.5	24.7	3.99
			Inner area	26.5	5.3	0.60

Data were averaged from indicated sample size (n). The field (80 cm in diameter circle) was divided into two concentric circles: an outer area (10 cm ring along the wall) and an inner circular area (60 cm in diameter)

**Table 3:** Quantitative ultrastructural analysis of CA1 synapses

	Control	ELKS cKO
Synapse density (per 100 μm^2^)	37.3 ± 1.12	38.7 ± 1.23
Presynaptic terminal area (nm^2^)	0.177 ± 0.017	0.190 ± 0.017
Size of active zone (nm)	182.0 ± 3.22	180.3 ± 6.82
Docked synaptic vesicle (per 100 nm active zone)	1.64 ± 0.256	1.86 ± 0.203

Images were obtained from the striatum radiatum of the hippocampal CA1 region. Data are presented as mean ± SEM, determined by the values from more than 100 synapses from each of 3 individual animals (control and ELKS cKO)

### Generation of CAST KO; ELKS cKO in the forebrain led to high mortality of new born pups

**Fig. 2:**
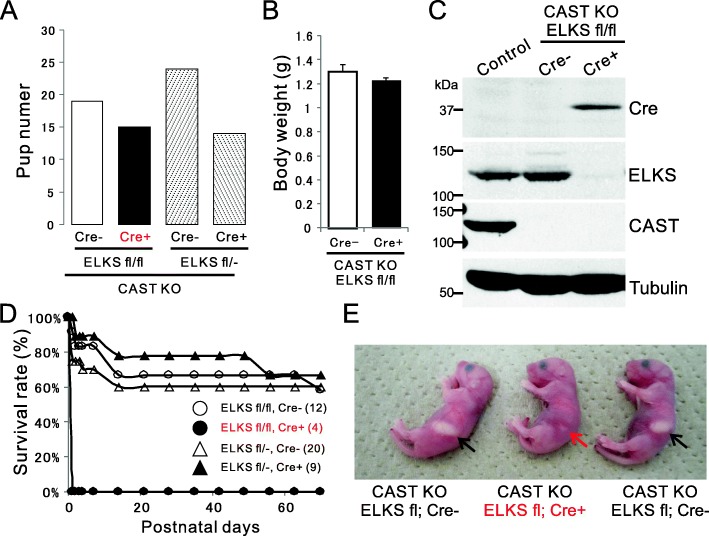
High mortality of CAST KO; ELKS cKO newborn pups. **a** To generate the CAST/ELKS dKO in the forebrain, ELKS cKO mice (via CaMKII-Cre) were crossed with global KO of CAST. Breeding pairs of CAST KO; ELKS^fl/fl^; CaMKII-Cre^−/−^ with CAST KO; ELKS^fl/−^; CaMKII-Cre^+/−^ resulted in pups with four different genotypes: ELKS fl with Cre- (19 of 72 P0–1 pups) and Cre + (15), or ELKS fl/− with Cre- (24) and Cre + (14). Chi-squared test, *p* = 0.33 **b** The newborn CAST/ELKS dKO (CAST KO; ELKS flox; CaMKII Cre+) could not be distinguished from other genotypes and the body weight was not altered. n = 3 for each genotype. **c** Expression of CAST and ELKS in the forebrain at P0–1 was analyzed by western blot using the indicated antibodies. In dKO pups, Cre expression depleted the ELKS. **d** The survival curve indicates high mortality of CAST/ELKS dKO pups at P0–1. Data shows the mean survival rate of each mouse genotype (numbers are indicated in parentheses). **e** Monitoring of newborn pups showed all were reddish and appeared normal, suggesting functional cardiovascular and respiratory systems. However, dKO pups had no milk in their stomachs, while other genotypes did (arrows)

**Fig. 3:**
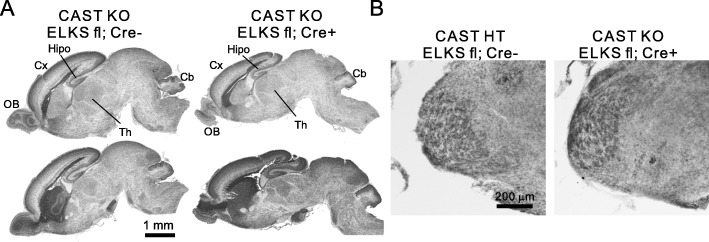
Major brain anatomy and the sensory network were normal in CAST/ELKS dKO pups. **a** Hematoxylin and eosin staining of P0–1 pup parasagittal whole-brains showed ELKS depletion in CAST KO mice had no conspicuous effect on brain anatomy development in the olfactory bulb (OB), cortex (Cx), hippocampus (Hipo), thalamus (Th), and cerebellum (Cb). **b** Cytochrome oxidase histochemistry at the spinal trigeminal nucleus of control (CAST HT; ELKS flox) and CAST/ELKS dKO (CAST KO; ELKS flox; CaMKII Cre+). Distinct patches, corresponding to the whiskers were present in both groups of mice

Separate CAST and ELKS deletion in forebrain synapses showed that CAST predominantly works in neurotransmitter release [11, 12]. However, because ELKS expression was upregulated in CAST KO [12], and presynaptic machinery proteins can complement each other’s roles [4, 5], here we generated CAST/ELKS dKO mice by crossing ELKS flox with CaMKII Cre^+/−^ mice with the CAST KO mice. From the CAST KO; ELKS^flox/flox^; CaMKII Cre^−/−^ with CAST KO; ELKS^flox/−^; CaMKII Cre^+/−^ breeding pairs, genotype distribution of offspring was as follows: CAST KO; ELKS^flox/flox^; CaMKII Cre^−/−^ (26%), CAST KO; ELKS^flox/flox^; CaMKII Cre^+/−^ (dKO, 21%), CAST KO; ELKS^flox/−^; CaMKII Cre^−/−^ (33%), and CAST KO; ELKS^flox/−^; CaMKII Cre^+/−^ (19%), corresponding to the expected Mendelian inheritance (Fig. 2a). Body weight of newborn pups did not significantly differ between CAST KO and dKO mice (Fig. 2b). Furthermore, depletion of CAST and ELKS was confirmed by immunoblotting (Fig. 2c). Although the offspring were in accordance with Mendelian law, we found that the dKO mice frequently died immediately after birth, and no dKO pup could survive more than one day (Fig. 2d). The pups were all reddish and appeared normal, suggesting functional cardiovascular and respiratory systems (Fig. 2e). However, unlike the other genotypes, the dKO pups had no milk in their stomachs. These results suggest that the CAST/ELKS dKO in the forebrain caused a defect in exploring and/or suckling behavior. Sensory signals for temperature, touch, and pain from the ipsilateral portion of a face pass through the spinal trigeminal nucleus to the contralateral thalamus, and are then relayed to primary motor cortex via sensory cortex [15]. Hematoxylin and eosin staining at postnatal day 0 (P0) brain showed that the olfactory bulb, cortex, hippocampus, thalamus, cerebellum, cortical laminae, and the pyramidal and granule cells in the hippocampus were correctly arranged in both groups, suggesting that histological features were similar between CAST KO and dKO (Fig. 3a). To confirm an intact brain-stem sensory tract, we performed cytochrome oxidase histochemistry on the CAST/ELKS dKO pups, successfully detecting the barrelette-like patches that represent the homeomorphic pattern of rodent whiskers in the spinal trigeminal nucleus (Fig. 3b). In a previous report, a similar phenotype in that the mutant mice died within one day after birth was reported from knockout of the N-methyl-D-aspartate (NMDA) receptor subunit NR2B (NMDA ε2) which belongs to the family of ionotropic glutamate receptor on postsynaptic site [16]. This NR2B knockout was regarded to possess inadequate suckling movements in response to sensory input, and exhibited abnormal barrelette-like patches in the brainstem trigeminal complex [16]. Therefore, unlike the NR2B knockout pups, the CAST/ELKS dKO pups had intact sensory transmission from the face to the brain stem. However, the lack of CAST/ELKS expression in thalamic neurons projecting to the cortex, or in cortical neurons, might have disrupted neurotransmission linking sensory inputs to suckling behavior.

Excluding studies of retinal photoreceptors, previous research on CAST/ELKS deletion mutants examined their roles related to regulation of neurotransmitter release rather than the formation of synapses [6, 7, 9, 11–13]. In these studies, animals with CAST KO and/or ELKS cKO in the nervous system were viable and mostly fertile. Therefore, the current study is the first showing that CAST/ELKS-regulated neural transmission has an essential role in pup survival. However, the CaMKII promoter-induced Cre depletion removed ELKS from most forebrain neurons and ELKS expression was thus almost completely suppressed (Fig. 2c). This makes it difficult to investigate the responsible neurons or neural network governing initial suckling behavior. We hope that future investigations that make specific double deletion of CAST and ELKS in other mouse neurons will demonstrate the contribution of presynaptic release machinery in regulating sensory inputs related to sucking behavior in infants.

## Materials and methods

### Generation of forebrain specific ELKS cKO mice

Inducible forebrain-specific ELKS mutant mice were obtained by crossing *ELKS* ^*flox/flox*^ mice [9] with *CaMKII-Cre* mice carrying Cre recombinase under the control of the *CaMKII* promoter [14]. The *ELKS* ^*flox/flox*^ mice were further crossed with *CAST*^*−/−*^ [6] to generate *CAST*^*−/−*^*; ELKS* ^*flox/flox*^ mice. The mice derived from crossing *ELKS* ^*flox/flox*^ with *ELKS* ^*flox/flox*^*; CaMKII-Cre*^*+/−*^ mice and *CAST*^*−/−*^*; ELKS* ^*flox/flox*^ with *CAST*^*−/−*^*; ELKS* ^*flox/-*^*; CaMKII-Cre*^*+/−*^ mice were used for subsequent studies. Genotyping of ELKS cKO, CAST KO, and dKO mice by PCR was performed as described previously [7].

### Open field test

The open field test was conducted using a circular apparatus with gray walls (diameter: 80 cm; height: 45 cm) [17]. The floor of the field was divided into two concentric circles, with an inner 60-cm diameter circular region. The mice were allowed to freely explore the environment for 30 min. During this time, movements were recorded and analyzed with a video-computerized tracking system (SMART, Panlab SL).

### Immunoblotting

Forebrain homogenates (20 μg of protein) from adult control and ELKS cKO and from P0–1 CAST KO and CAST KO/ELKS cKO mice were analyzed using western blotting [18]. Briefly, after SDS-PAGE was performed on 10% polyacrylamide gels, proteins were transferred to PVDF membranes following standard procedures. The membranes were blocked with 5% (w/v) non-fat milk powder in TBST (25 mM Tris/HCl, pH 7.5, 150 mM NaCl, and 0.05% Tween 20), followed by an overnight incubation with primary antibodies; anti-Cre (Millipore, MAB3120), anti-ELKS [19], anti-CAST [20], and anti-tubulin (Oncogene, CP06). After washing with TBST, membranes were incubated with horseradish peroxidase-labeled secondary antibodies for 1 h. After washing, membranes were treated with ECL solution and the immunoreactive bands were detected on the films.

### Immunohistochemistry

Under deep pentobarbital anesthesia, mice were fixed transcardially with 4% paraformaldehyde and 10% picric acid in 0.1 M phosphate buffer (pH 7.4). Brain sections (thickness, 100 μm) were made with a Microslicer (DTK-1000 N, Dosaka), and incubated overnight with the following primary antibodies: anti-Cre, and anti-ELKS diluted in blocking solution (0.5% Carrageenan, 0.1% Triton X-100, 2.5% normal goat serum in PBS). The brain sections were further processed with appropriate Alexa Fluor-conjugated secondary antibodies for 1 h. Immunolabeled samples were viewed using a confocal laser microscope (FV1200, Olympus).

### Electron microscopy

Sample preparation for electron microscopy was described previously [7]. Briefly, deeply anesthetized mice were fixed in 2% paraformaldehyde and 2% glutaraldehyde in 0.1 M phosphate buffer (PB, pH 7.4), and hippocampal slices (thickness, 100 μm) were sectioned. After washing with PB, slices were further fixed with 2% osmium tetroxide, stained with 2–4% uranyl acetate, and embedded in epoxy resin (Durcupan ACM-Fluka, Sigma). Ultra-thin sections (thickness, 70 nm) were counter stained with uranyl acetate and lead citrate, and images were captured with an electron microscope (H-7500, Hitachi). Images were analyzed with Image-J according to previously described parameters [12].

### Cytochrome oxidase histochemistry

As described previously [16], neonatal pups were fixed by transcardial perfusion with 4% paraformaldehyde and 0.2% picric acid in 0.1 M PB, and decapitated. Brains were cryoprotected with 30% sucrose in PB and cut into 30-μm coronal sections through the spinal trigeminal nucleus. Cytochrome oxidase reactions were performed for 12 h at 37 °C in a solution containing 0.3 mg/ml of cytochrome C, 0.5 mg/ml of 3,3′-diamino-benzidine, and 45 mg/ml of sucrose in PB.

## Data Availability

The datasets used and/or analyzed during the current study are available from the corresponding author upon reasonable request.

## Abbreviations

*AZ*: Active zone
*CAST*: Cytomatrix at the active zone-associated structural protein
*CAZ*: Cytomatrix at the AZ
*KO*: Knockout
*NMDA*: N-methyl-D-aspartate
